# Erratum: Hołub et al. Predicting Breaststroke and Butterfly Stroke Results in Swimming Based on Olympics History. *Int. J. Environ. Res. Public Health* 2021, *18*, 6621

**DOI:** 10.3390/ijerph18168401

**Published:** 2021-08-09

**Authors:** Maciej Hołub, Arkadiusz Stanula, Jakub Baron, Wojciech Głyk, Thomas Rosemann, Beat Knechtle

**Affiliations:** 1 Institute of Sport Sciences, Jerzy Kukuczka Academy of Physical Education, 40-065 Katowice, Poland; m.holub@awf.katowice.pl (M.H.); a.stanula@awf.katowice.pl (A.S.); j.baron@awf.katowice.pl (J.B.); w.glyk@awf.katowice.pl (W.G.); 2 Institute of Primary Care, University of Zurich, 8091 Zurich, Switzerland; thomas.rosemann@usz.ch; 3 Medbase St. Gallen Am Vadianplatz, 9000 St. Gallen, Switzerland

## Error in Table

In the original article [1], there was a mistake in “**the table footer of Table 1. Summary of univariate linear regression for the results of the first and eighth place in the finals and the average time of the women’s and men’s finalists in the 100-m and 200-m breaststroke and butterfly stroke along with a prediction of times for the Tokyo Olympic Games in 2021**.” as published. 

“The last sentence in table footer “**3.2. 200 m Breaststroke**” does not belong to this table footer; it should be the heading of “3.2. 200 m Breaststroke”. The corrected table footer should be “Note: The analysis involved results achieved in the Olympics since 1972 (N = 12). F—value of one-way analysis of variance, df—degrees of freedom, r—Pearson correlation coefficient, r^2^—coefficient of determination, LL—lower limit, UL—upper limit. *—*p* < 0.001”.

“3.2. 200 m Breaststroke” in table footer changed to be subtitle “*3.2. 200 m Breaststroke*”; Subtitle “*3.2. 100 m Butterfly*” changed to be subtitle “*3.3. 100 m Butterfly*”; Subtitle “*3.3. 200 m Butterfly*” changed to be subtitle “*3.4. 200 m Butterfly*”. 

The authors apologize for any inconvenience caused and state that the scientific conclusions are unaffected. The original article has been updated.
